# Construction of a North American Cancer Survival Index to Measure Progress of Cancer Control Efforts

**DOI:** 10.5888/pcd14.170201

**Published:** 2017-09-14

**Authors:** Christopher J. Johnson, Hannah K Weir, Angela Mariotto, Reda Wilson, Diane Nishri

**Affiliations:** 1 Cancer Data Registry of Idaho, Idaho Hospital Association, Boise, Idaho; 2 Division of Cancer Prevention and Control, Centers for Disease Control and Prevention, Atlanta, Georgia; 3 Division of Cancer Control and Population Sciences, National Cancer Institute, Bethesda, Maryland; 4 Cancer Care Ontario, Toronto, Ontario, Canada

## Abstract

**Introduction:**

Population-based cancer survival data provide insight into the effectiveness of health care delivery. Comparing survival for all cancer sites combined is challenging, because the primary cancer site and age distribution of patients may differ among areas or change over time. Cancer survival indices (CSIs) are summary measures of survival for cancers of all sites combined and are used in England and Europe to monitor temporal trends and examine geographic differences in survival. We describe the construction of the North American Cancer Survival Index and demonstrate how it can be used to compare survival by geographic area and by race.

**Methods:**

We used data from 36 US cancer registries to estimate relative survival ratios for people diagnosed with cancer from 2006 through 2012 to create the CSI: the weighted sum of age-standardized, site-specific, relative survival ratios, with weights derived from the distribution of incident cases by sex and primary site from 2006 through 2008. The CSI was calculated for 32 registries for all races, 31 registries for whites, and 12 registries for blacks.

**Results:**

The survival estimates standardized by age only versus age-, sex-, and site-standardized (CSI) were 64.1% (95% confidence interval [CI], 64.1%–64.2%) and 63.9% (95% CI, 63.8%–63.9%), respectively, for the United States for all races combined. The inter-registry ranges in unstandardized and CSI estimates decreased from 12.3% to 5.0% for whites, and from 5.4% to 3.9% for blacks. We found less inter-registry variation in CSI estimates than in unstandardized all-sites survival estimates, but disparities by race persisted.

**Conclusions:**

CSIs calculated for different jurisdictions or periods are directly comparable, because they are standardized by age, sex, and primary site. A national CSI could be used to measure temporal progress in meeting public health objectives, such as Healthy People 2030.

## Introduction

Progress in meeting cancer control objectives can be measured by using a combination of statistics on cancer incidence, population-based survival, and mortality (1–3). Comparing survival, in particular, among geographic areas and over time can aid in the understanding of inequities and changes in the quality and effectiveness of health care provided to population groups of people diagnosed with cancer (4). However, interpreting the results of comparisons of survival proportions for all cancer sites combined is challenging when distributions by age, sex, and primary cancer site differ by geographic area or change over time.

Relative survival is a measure of excess mortality among cancer patients and is a useful statistic for comparing trends in survival over time or between different geographic areas, because it endeavors to remove the effects of competing causes of death. Relative survival statistics may be age-standardized to account for different age structures in the patient populations being compared (5,6). However, to make comparisons of relative survival for all cancer sites combined requires adjusting for the case-mix of primary cancer sites. European Cancer Registry Based Study on Survival and Care of Cancer Patients (EUROCARE) researchers and others have performed age and case-mix adjustments for England and Europe to compare survival proportions between nations and among local areas and to monitor temporal trends (7–10).

In this article, we describe construction of the North American Cancer Survival Index (CSI), which standardizes for age, sex, and primary cancer site, and compare unstandardized and primary-site–standardized survival proportions for all cancer sites combined, by registry jurisdiction and race. We demonstrate its use in comparative analysis of registry- and race-specific survival and describe its use as a baseline measure for monitoring progress over time in cancer control efforts and in meeting public health objectives related to improving early cancer diagnosis and access to timely, evidenced-based treatment.

## Methods

### Data source

All population-based cancer registries in the United States and Canada are members of the North American Association of Central Cancer Registries (NAACCR). Beginning with data from 1996, NAACCR has produced the *Cancer in North America* (CINA) reports (https://www.naaccr.org/cancer-in-north-america-cina-volumes/) of cancer incidence and mortality rates in the United States and Canada. Beginning in 2015, NAACCR asked member registries to provide follow-up data for the purpose of reporting survival proportions. For registries to be included in the survival analysis described in this article, they needed to provide consent, meet CINA incidence criteria for all relevant years (11), and either meet the National Cancer Institute's Surveillance, Epidemiology, and End Results (SEER) standards for follow-up (12) or ascertain deaths through our study’s cutoff date, December 31, 2012, through linkages with state death records and the National Death Index (13). Survival data were provided by 36 US registries (31 states and 5 metropolitan areas) on more than 6.6 million cancers diagnosed from 2006 through 2012 (6). To avoid double-counting in the survival estimates, data from metropolitan area registries in California and Georgia were not included in the United States combined statistics. The data set included malignant cases as defined by the SEER behavior recode for analysis (14) for people aged 15 to 99 years diagnosed from 2006 through 2012. 

### Statistical analysis

We excluded incident cases that were reported solely via death certificates or autopsy. For registries conducting active follow-up, alive cases with no survival time were excluded from analysis. By using SEER 2007 Multiple Primary and Histology Coding Rules (15), we allowed for multiple primary cancers to be included for each patient, but only the first applicable record per patient was included in each survival estimate.

SEER*Stat software version 8.2.1 (Information Management Services, Inc) was used to perform survival calculations (16). The survival duration in months was calculated on the basis of complete dates. For registries meeting SEER follow-up standards (SEER registries plus Montana and Wyoming), the survival duration for alive patients was calculated through the date of last contact (or study cutoff, if earlier). For the remaining registries, survival duration for alive patients was calculated through December 31, 2012, with all patients not known to be dead presumed to be alive on this date (17).

Sixty-month age-standardized relative survival ratios (RSRs) were calculated by using the actuarial method on monthly intervals. We calculated relative survival by using the Ederer II method to compute expected survival (18). Expected survival was estimated from life tables matched to cancer patients by age, sex, year, geographic area, race, and socioeconomic status (19). Cases were censored at an achieved patient age of 100 years.

### Cancer survival index

The construction of the CSI was described in the technical notes of *Cancer Survival in the United States and Canada 2006–2012* (6). Briefly, the CSI is the weighted sum of the age-standardized site-specific RSRs, with the weights derived from the proportionate distribution of North American incidence counts for diagnosis years 2006 through 2008 as reported for the November 2014 Call for Data (Table 1). This range of years was selected, because the incidence data for these years are more mature in terms of reporting delay than more recent years. Case counts to derive the weights were limited to malignant behavior and urinary bladder in situ neoplasms among patients aged 15 years or older, and SEER metropolitan-area registries were excluded to avoid double-counting of incident cases for their respective states.

**Table 1:** Weights Used In Case-Mix Standardization of Estimated Relative Survival Ratios for the North American Cancer Survival Index^a^

Primary Cancer Site	Sex-Specific		Male and Female Patients Combined		Both Sexes^b^
	Male	Female	Male	Female	
Brain and other nervous system	1.360	1.203	0.710	0.575	1.285
Breast	0.242	29.264	0.126	13.990	14.116
Cervix uteri	0.000	1.806	0.000	0.864	0.864
Colon and rectum	9.981	10.287	5.210	4.918	10.128
Corpus and uterus, not otherwise specified	0.000	5.943	0.000	2.841	2.841
Esophagus	1.601	0.485	0.835	0.232	1.067
Hodgkin lymphoma	0.584	0.533	0.305	0.255	0.560
Kidney and renal pelvis	3.948	2.675	2.061	1.279	3.339
Larynx	1.272	0.352	0.664	0.168	0.832
Leukemia	2.803	2.284	1.463	1.092	2.555
Liver and intrahepatic bile duct	1.945	0.837	1.015	0.400	1.416
Lung and bronchus	14.788	13.787	7.718	6.591	14.309
Melanoma of the skin	4.418	3.591	2.306	1.717	4.023
Mesothelioma	0.326	0.103	0.170	0.049	0.219
Myeloma	1.327	1.206	0.693	0.576	1.269
Non-Hodgkin lymphoma	4.212	3.982	2.198	1.903	4.102
Oral cavity and pharynx	3.171	1.521	1.655	0.727	2.382
Ovary	0.000	3.067	0.000	1.466	1.466
Pancreas	2.438	2.675	1.272	1.279	2.551
Prostate	29.321	0.000	15.304	0.000	15.304
Stomach	1.718	1.179	0.897	0.564	1.461
Testis	1.028	0.000	0.537	0.000	0.537
Thyroid	1.138	3.945	0.594	1.886	2.480
Urinary bladder	6.585	2.367	3.437	1.131	4.568
Other	5.793	6.908	3.024	3.302	6.326
Total	100	100	100		100

a Weights were derived from the proportionate distribution of North American Association of Central Cancer Registries incident cases for people aged ≥15 y, by sex and primary cancer site for diagnosis years 2006 through 2008.

b Both sexes’ weights should be used only when separate survival estimates for male and female patients are not available. The resulting weighted survival measure will be adjusted for site mix, but not for the proportion of male and female patients.

Separate sets of weights were used for male patients, female patients, and male and female patients combined. Let *S_i_* be the age-standardized, site-specific relative survival ratio estimate and *W_i_* be the proportion of the sex-specific incidence counts for site category *i*. The cancer survival index (CSI) and its standard error are:


*CSI* = *∑S_i_W_i_*

*standard error* (*CSI*) = [*∑standard error *(*S_i_*)^2^*W_i_*^2^]^1/2^

Ninety-five percent confidence intervals were calculated by using the normal approximation on the logarithmic scale:

Lower limit = CSI / exp[1.96 * standard error (CSI) / CSI]

Upper limit = CSI * exp[1.96 * standard error (CSI) / CSI]

For the purpose of this analysis, 2 sets of statistics for all cancer sites combined are presented. The first is labeled “all sites” and shows the age-standardized RSRs for all cancer sites combined using the International Cancer Survival Standard age standard 1 (5). The all sites survival statistics reflect the primary site distribution in each registry jurisdiction. The second statistics set is labeled CSI and shows a composite cancer survival index. In calculating the CSI, if a site-specific age-standardized RSR was not available for a registry jurisdiction, such as for rare cancers in smaller populations, the estimate was replaced with the RSR for the United States. For race-specific CSI calculations, the replacement used the RSR for the United States for that race. More cases are included in the CSI than in the all sites set because the all sites statistics set includes only one case per person, but a person could contribute one case each to many of the individual site categories in the CSI (20). Confidence intervals for the CSI can be narrower than for the all sites statistics set because of the national replacement data and the larger numbers of cases. The standard error of the CSI was not adjusted for the potential inclusion of more than one case per person because these cases made up only 4% of the total. If more than 30% of the site-specific age-standardized RSR estimates were unavailable for a registry jurisdiction and were replaced with that of the country, the CSI estimate was suppressed to avoid unduly biasing the results.

We used funnel plots (Figure) to show 5-year RSR estimates (vertical axes) plotted against the precision of the estimates (horizontal axes) (21). Precision was calculated as the inverse of the variance of the survival estimates. The horizontal solid lines in the figures are the values for the US combined estimates. The control limits were established by using the range of standard errors from the registry-specific survival estimates and are shown as the lower and upper percentile limits of the standard Normal distribution (*z* = 1.96 for 95% control limits and *z* = 3.09 for 99.8% control limits) around US combined estimates. Funnel plots help identify divergent estimates better than rankings but do not test for multiple comparisons.

**Figure:**
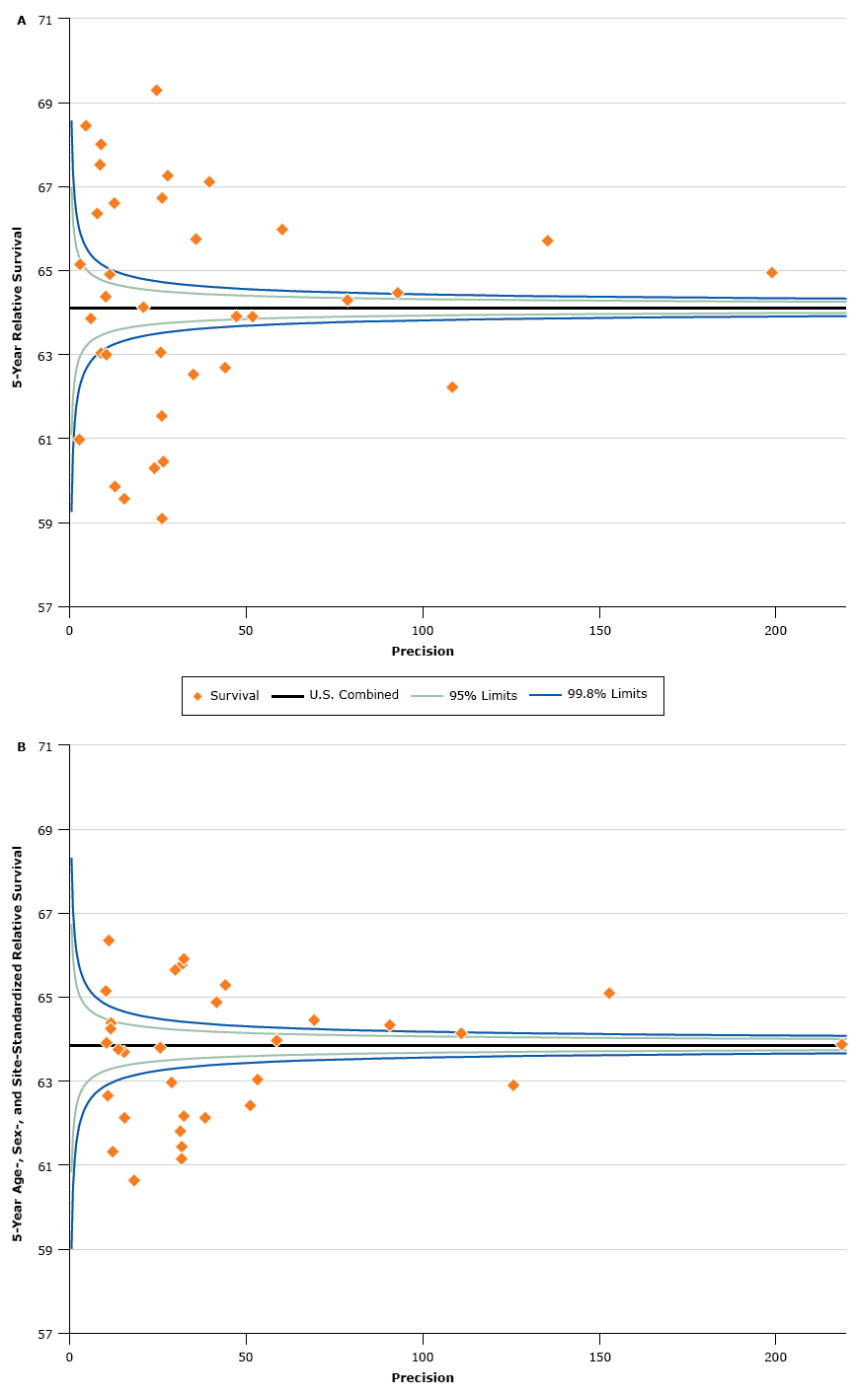
Graph a shows a funnel plot of 5-year age-standardized relative survival ratios for all cancer sites combined for men and women diagnosed with cancer from 2006 through 2012 and followed up on December 31, 2012. Graph b shows a funnel plot of 5-year age-, sex-, and site-standardized relative survival ratios, calculated by using the North American Cancer Survival Index (CSI), for men and women diagnosed with cancer from 2006 through 2012 and followed up on December 31, 2012. Graphs a and b show funnel plots of 5-year relative survival plotted against precision, such that low-precision estimates are on the left side, and high-precision estimates are on the right side. Precision was calculated as the inverse of the variance of the survival estimates. The control limits were established by using the range of standard errors from the registry-specific survival estimates and are shown as the lower and upper percentile limits of the standard Normal distribution (*z* = 1.96 for 95% control limits and *z* = 3.09 for 99.8% control limits) around US combined estimates. Graph a shows the dramatic variation in estimates of relative survival by registry jurisdiction for all sites combined. Graph b shows substantially less variation using the CSI, which is standardized by age, sex, and cancer-site mix, than in using the all sites combined statistics set. The survival estimates in graph b are overall much closer to the line for the United States combined. The figures are a graphic representation of the data in Table 2 that use a function of the standard error for precision.

## Results

The CSI cancer survival estimate was 63.9 for the United States combined (Table 2), 64.4 for US whites, and 55.9 for US blacks (Table 3). We found substantially less interjurisdictional variation in CSI survival estimates compared with survival estimates from the all sites statistics set. All sites RSRs varied from 59.1 in Kentucky to 71.1 in Utah, whereas CSI RSRs varied from 60.6 in Mississippi to 66.3 in New Hampshire (Table 2; Figure). The state with the biggest negative difference between all sites and CSI estimates was Utah (71.1, all sites vs 64.3, CSI = −6.8), and the state with the biggest positive difference between the all sites and CSI estimates was Kentucky (59.1, all sites vs 62.2, CSI = 3.1). Other states with large negative differences from the all sites estimates to CSI estimates were Colorado and Idaho, and the other state with a large increase from all sites to CSI estimates was West Virginia. Registry jurisdictions with all-races-combined CSI values greater than 65.0 were California–Greater Bay Area, Colorado, Connecticut, New Hampshire, New York, Rhode Island, and the Seattle/Puget Sound SEER registry. The standard error of the jurisdiction-specific point estimates was 47% lower for the CSI as compared with that for the all sites combined statistics.

**Table 2:** Five-Year Age-Standardized Relative Survival Ratios (RSRs) for All Sites Combined and North American Cancer Survival Index (CSI)^a^ for US Cancer Patients Aged 15 to 99 Years Diagnosed From 2006–2012

Cancer Registry Jurisdiction	All Sites RSR (95% Confidence Interval)	CSI RSR (95% Confidence Interval)
United States combined	64.1 (64.1–64.2)	63.9 (63.8–63.9)
Alabama	60.5 (60.1–60.8)	61.4 (61.1–61.8)
Alaska	61.0 (59.8–62.2)	—
Arizona	62.5 (62.2–62.9)	62.1 (61.8–62.4)
California	65.0 (64.8–65.1)	63.9 (63.7–64.0)
California, Greater Bay Area	67.1 (66.8–67.4)	65.3 (65.0–65.6)
California, Los Angeles	63.9 (63.6–64.2)	63.0 (62.8–63.3)
Colorado	69.3 (68.9–69.7)	65.8 (65.4–66.1)
Connecticut	66.7 (66.3–67.1)	65.7 (65.3–66.0)
Georgia	62.7 (62.4–63.0)	62.4 (62.1–62.7)
Georgia, Atlanta	66.6 (66.1–67.2)	63.7 (63.2–64.2)
Hawaii	63.0 (62.4–63.7)	62.7 (62.1–63.3)
Idaho	67.5 (66.8–68.2)	63.9 (63.3–64.5)
Illinois	64.3 (64.1–64.5)	64.3 (64.1–64.5)
Iowa	64.1 (63.7–64.6)	63.8 (63.4–64.2)
Kentucky	59.1 (58.7–59.5)	62.2 (61.8–62.5)
Louisiana	60.3 (59.9–60.7)	61.2 (60.8–61.5)
Maine	64.4 (63.8–65.0)	64.4 (63.8–65.0)
Michigan, Detroit	63.1 (62.7–63.4)	63.0 (62.6–63.3)
Mississippi	59.6 (59.1–60.1)	60.6 (60.2–61.1)
Montana	63.9 (63.0–64.7)	—
Nebraska	64.9 (64.3–65.5)	63.8 (63.2–64.3)
New Hampshire	68.0 (67.3–68.7)	66.3 (65.8–66.9)
New Jersey	66.0 (65.7–66.2)	64.5 (64.2–64.7)
New Mexico	63.0 (62.4–63.6)	61.3 (60.8–61.9)
New York	65.7 (65.5–65.9)	65.1 (64.9–65.3)
North Carolina	63.9 (63.6–64.2)	64.0 (63.7–64.2)
North Dakota	68.4 (67.5–69.4)	—
Pennsylvania	64.5 (64.3–64.7)	64.1 (64.0–64.3)
Rhode Island	66.4 (65.6–67.1)	65.1 (64.5–65.8)
South Carolina	61.5 (61.2–61.9)	61.8 (61.5–62.2)
Texas	62.2 (62.0–62.4)	62.9 (62.7–63.1)
Utah	71.1 (70.5–71.7)	64.3 (63.7–64.8)
Washington, Seattle	67.3 (66.9–67.6)	65.9 (65.6–66.3)
West Virginia	59.9 (59.3–60.4)	62.1 (61.6–62.6)
Wisconsin	65.8 (65.4–66.1)	64.9 (64.6–65.2)
Wyoming	65.1 (64.0–66.3)	—

Abbreviation: —, not calculable.

a The CSI is the weighted sum of the age-standardized site-specific RSRs, with the weights derived from the proportionate distribution of North American incidence counts for diagnosis years 2006 through 2008.

**Table 3:** Five-Year Age-Standardized Relative Survival Ratios (RSRs) for All Sites Combined and North American Cancer Survival Index (CSI)^a^ for US Cancer Patients Aged 15 to 99 Years Diagnosed From 2006 Through 2012, By Race

Cancer Registry Jurisdiction	White Race		Black Race	
	All Sites RSR (95% CI)	CSI (95% CI)	All Sites RSR (95% CI)	CSI (95% CI)
United States combined	64.7 (64.7–64.8)	64.4 (64.4–64.5)	56.8 (56.6–57.0)	55.9 (55.6–56.1)
Alabama	61.2 (60.7–61.6)	62.6 (62.2–63.0)	55.3 (54.5–56.2)	—
Alaska	63.4 (62.0–64.8)	—	60.0 (52.0–67.1)	—
Arizona	62.3 (61.9–62.6)	62.0 (61.7–62.3)	54.9 (52.5–57.2)	—
California	65.2 (65.1–65.4)	64.0 (63.9–64.2)	57.1 (56.6–57.7)	55.7 (55.0–56.4)
California, Greater Bay Area	68.8 (68.4–69.1)	66.0 (65.6–66.3)	57.2 (55.9–58.4)	—
California, Los Angeles	64.9 (64.5–65.2)	63.9 (63.6–64.2)	56.1 (55.2–57.0)	—
Colorado	68.3 (67.9–68.7)	65.4 (65.0–65.8)	62.4 (60.0–64.7)	—
Connecticut	67.0 (66.6–67.4)	65.9 (65.6–66.3)	59.6 (57.9–61.2)	—
Georgia	64.1 (63.7–64.4)	64.0 (63.7–64.3)	58.1 (57.5–58.8)	55.5 (54.8–56.3)
Georgia, Atlanta	70.1 (69.5–70.8)	66.3 (65.7–66.9)	60.0 (58.9–61.0)	—
Hawaii	68.2 (67.0–69.5)	—	65.7 (55.2–74.3)	—
Idaho	67.2 (66.5–67.9)	63.8 (63.1–64.4)	62.9 (47.8–74.8)	—
Illinois	64.8 (64.6–65.0)	64.8 (64.6–65.0)	56.7 (56.1–57.4)	56.5 (55.8–57.1)
Iowa	64.0 (63.5–64.4)	63.7 (63.3–64.1)	53.4 (49.3–57.2)	—
Kentucky	59.3 (58.9–59.6)	62.3 (62.0–62.7)	53.8 (52.2–55.4)	—
Louisiana	62.6 (62.1–63.1)	63.1 (62.7–63.5)	54.2 (53.4–55.0)	53.9 (53.1–54.7)
Maine	64.0 (63.4–64.6)	64.2 (63.6–64.8)	64.3 (52.0–74.2)	—
Michigan, Detroit	64.5 (64.1–65.0)	64.3 (63.9–64.7)	56.1 (55.2–56.9)	55.4 (54.7–56.2)
Mississippi	61.8 (61.2–62.4)	63.2 (62.6–63.7)	54.2 (53.2–55.1)	53.0 (52.1–53.9)
Montana	64.6 (63.8–65.4)	—	—	—
Nebraska	64.8 (64.2–65.4)	63.7 (63.1–64.2)	55.5 (51.9–59.0)	—
New Hampshire	68.0 (67.4–68.7)	66.4 (65.8–67.0)	63.6 (50.7–74.0)	—
New Jersey	67.1 (66.8–67.4)	65.4 (65.2–65.7)	56.3 (55.4–57.1)	54.0 (53.2–54.9)
New Mexico	63.3 (62.7–63.9)	61.4 (60.8–62.0)	53.1 (47.4–58.5)	—
New York	66.4 (66.2–66.6)	65.7 (65.6–65.9)	59.5 (59.1–60.0)	56.6 (56.1–57.2)
North Carolina	65.1 (64.8–65.3)	65.1 (64.8–65.4)	58.4 (57.7–59.0)	56.9 (56.2–57.6)
North Dakota	69.1 (68.1–70.0)	—	—	—
Pennsylvania	64.5 (64.3–64.7)	64.3 (64.1–64.5)	55.8 (55.0–56.6)	55.7 (55.0–56.5)
Rhode Island	66.2 (65.4–66.9)	65.1 (64.5–65.8)	57.6 (53.2–61.7)	—
South Carolina	62.7 (62.3–63.1)	63.2 (62.8–63.6)	55.9 (55.1–56.8)	54.5 (53.7–55.3)
Texas	62.8 (62.6–63.0)	63.4 (63.2–63.6)	54.5 (53.9–55.1)	54.9 (54.2–55.5)
Utah	71.6 (70.9–72.2)	64.5 (63.9–65.1)	57.7 (48.9–65.5)	—
Washington, Seattle	68.0 (67.7–68.4)	66.2 (65.8–66.6)	59.8 (57.5–62.1)	—
West Virginia	59.9 (59.3–60.4)	62.2 (61.7–62.7)	54.6 (51.0–58.1)	—
Wisconsin	66.6 (66.2–66.9)	65.3 (65.0–65.7)	55.9 (53.9–57.8)	—
Wyoming	65.4 (64.3–66.6)	—	58.0 (43.4–70.2)	—

Abbreviations: —, not calculable; CI, confidence interval.

a The CSI is the weighted sum of the age-standardized site-specific RSRs, with the weights derived from the proportionate distribution of North American incidence counts for diagnosis years 2006 through 2008.

Estimates outside of the control limits for the all sites and CSI statistics sets are recognized as differing from the respective combined US values, with estimates below the lower control limit considered to be low outliers, and estimates above the upper control limit considered to be high outliers. For the all sites combined statistics there were 12 registry-specific values below the lower control limit and 13 registry-specific values above the upper control limit (Figure a). For the CSI there were 13 registry-specific values below the lower control limit and 10 registry-specific values above the upper control limit (Figure b).

For whites, all sites RSRs varied from 59.3 in Kentucky to 71.6 in Utah, while the CSI RSRs varied from 61.4 in New Mexico to 66.4 in New Hampshire (Table 3). The state with the largest negative difference between the all sites and CSI estimates among whites was Utah (−7.0), and the state with the largest positive difference between the all sites and CSI estimates among whites was Kentucky (3.1).

For blacks in the 12 registries for which the CSI could be calculated (California, Georgia, Illinois, Louisiana, Michigan [metropolitan Detroit], Mississippi, New Jersey, New York, North Carolina, Pennsylvania, South Carolina, and Texas), the all sites RSRs varied from 54.2 in Mississippi to 59.5 in New York, while the CSI RSRs varied from 53.0 in Mississippi to 56.9 in North Carolina (Table 3). The registry with the largest negative difference between the all sites and CSI estimates was New York (−2.9). Texas had the largest positive difference between the all sites and CSI estimates (0.3).

In the same 12 registries for which the index could be calculated for blacks, the CSI RSRs for whites varied from 63.1 in Louisiana to 65.7 in New York. Although the within-race ranges in CSI values for the 12 registries were 2.7 (whites) and 3.9 (blacks), the median white–black differences in CSI values for the 12 registries were 8.7 for male and female patients combined, 8.2 for male patients, and 9.4 for female patients.

## Discussion

Cancer survival varies widely by age, sex, and site of the primary cancer. To compare overall cancer survival among registry jurisdictions, it is necessary to adjust for all 3 factors. In this article, we described construction of the North American CSI that was first used in the inaugural *CINA Survival* report and is, to our knowledge, the first set of site-mix adjusted cancer survival estimates for the United States (6). As expected, CSI ranges were narrower than the age-standardized all sites RSR estimates, which include different proportions of highly fatal cancers by registry jurisdiction (Figure).

The CSI is a summary measure of overall cancer survival and is intended to quantify and communicate disparities in cancer survival by race and across registry jurisdictions and to monitor progress in cancer survival over time. CSI statistics are directly comparable between registry jurisdictions and over time because they are standardized by age, sex, and primary-cancer–site distribution. This type of index has been suggested for use as an indicator for cancer control (7,22). EUROCARE routinely publishes age and case-mix standardized survival estimates that offer comparisons by country (8). Recently, the CSI has been used to demonstrate improvement in both short-term and long-term survival from all cancers combined over a 40-year period in England and Wales (22). The comparison of CSI estimates could be useful to policy makers, cancer control professionals and researchers, and other partners in population-based cancer control efforts in the United States. Although age and site-mix adjusted relative survival measures may be informative of a registry jurisdiction’s performance in cancer control, the indicator values may not be easily interpreted clinically (7).

Summary measures such as the CSI offer brevity at the expense of the detail that may be found in site-specific survival estimates. As with age-adjusted incidence rates versus age-specific rates or a stock market index versus individual stock prices, the value of the CSI is in its economy for an overview of broad patterns. Likewise, 5-year relative survival is a commonly published metric (23) but may not be the best duration to measure cancer control performance for each cancer site. Comparisons among states may be different with application of the CSI weights to different survival durations. Ideally, the CSI can be used in conjunction with site-specific survival estimates and incidence rates but should be considered superior to the all sites RSRs for comparing health systems performance among registry jurisdictions.

We recommend that the weights for calculating CSI estimates for male and female patients combined and separately be used to calculate age, sex, and site-standardized RSRs for North American registry jurisdictions when a one-number summary for overall patterns of cancer survival is desired. The right-most column in Table 1 shows weights for both sexes, which were not discussed in this article, but were included for completeness. Weights for both sexes should be used only when separate survival estimates for male and female patients are not available. The resulting weighted survival measure will be adjusted for site mix but not for the proportion of male patients and female patients diagnosed with cancer.

Variation in survival by registry catchment area can be due to several factors, including but not limited to differences in demographic characteristics related to race, ethnicity, and socioeconomic status; cancer screening rates and overdiagnoses associated with screening, which affect stage distributions; access to and quality of care; and cancer registration practices that affect case ascertainment, dates of diagnosis, and follow-up (24). The 3 states with larger negative difference from the all sites estimate to the CSI estimate (Colorado, Idaho, Utah) have lower historic smoking rates than other states, which portend a lower proportion of highly fatal cancers (25). The 2 states with the largest positive difference from the all sites to the CSI estimates (Kentucky and West Virginia) have higher historic smoking rates (25).

Each of the areas with all-races-combined CSI values greater than 65.0 has higher socioeconomic status than other states as measured by median income (26). Storm et al found that adjustment for case-mix is important in comparisons of relative survival across countries, and suggested additional patient characteristics such as stage, comorbidity, and risk factors might further explain such differences (9). An area for future consideration is the correlation of the North American CSI with measures of state, province, and territory-level screening and risk factor profiles, socioeconomic status, and health care access.

Our results show stark and consistent differences in survival by race for many cancer sites in the United States, a finding seen also in the first CONCORD Programme study and the latest SEER Cancer Statistics Review (23,27). Findings from our study show that sizable differences in cancer survival by race remain in the United States after adjusting for age, sex, and case mix. Of note, the CSI values for blacks spanned a narrow range from 53.0 (Mississippi) to 56.9 (North Carolina), and the disparity between whites and blacks varied little by registry. The CSI can be used to monitor progress toward eliminating disparities by race in the United States.

This study has several limitations. First, because age, sex, and case-mix standardized measures require estimates for each combination, CSI values could be calculated for only 12 of 36 US registry areas for blacks. Approximately 30,000 cases are necessary to calculate the CSI. Second, in registries for which survival time was calculated using the “presumed alive” method, survival may be biased upwards (28). However, 4 of the 5 highest CSI values among whites were in SEER registries, so this concern may not be problematic. Third, the CINA Survival reports released to date were not able to use life tables stratified by Hispanic ethnicity. Pinheiro et al have shown that in SEER data, Hispanics and Asians are more likely to have incomplete follow-up than non-Hispanic whites or blacks, and those with worse prognoses are more likely to have incomplete follow-up than those with better prognoses (29). This factor may have affected CSI values for states with high percentages of Hispanic residents, such as New Mexico and Texas. In addition, the life tables available for calculating expected survival may not reflect all factors contributing to variation in all-cause mortality, such as smoking. Finally, the US combined survival statistics may not be representative of the total national population because not all states were included.

Although the ranges in CSI values are narrower than their unadjusted variants, large disparities in cancer survival remain between blacks and whites in the United States. This summary survival measure is appropriate for interjurisdictional survival comparisons in the United States and as a baseline for monitoring progress over time in population-based cancer control efforts and in meeting public health objectives directed toward improving early diagnosis and access to evidenced-based treatment. For example, the US Department of Health and Human Services has begun planning for Healthy People 2030, scheduled for release in 2020 (30). The North American CSI, using the weights described in this article, could be used to measure progress in meeting the Healthy People objective related to cancer survival from the present through 2030.
